# Advancements in biochar nanocomposites: multifunctional platforms for sustainable energy storage and conversions

**DOI:** 10.1039/d6na00040a

**Published:** 2026-05-01

**Authors:** Monika Dubey, Piyush Kuchhal, Ranjit Kumar

**Affiliations:** a Department of Electrical and Electronics Engineering, University of Petroleum and Energy Studies Bidholi Dehradun Uttarakhand 248007 India; b Center for Advanced Materials, Department of Chemical Engineering, Shiv Nadar Institution of Eminence Delhi NCR – 201314 India ranjit.kumar@snu.edu.in

## Abstract

The increasing demand for renewable energy sources has intensified the need for sustainable, low-cost and efficient energy storage and conversion materials. Biochar is a carbonaceous solid obtained from biomass pyrolysis. It has recently attracted interest as an electrochemical material due to its tunable porosity, surface chemistry and renewable origin. When combined with nanoscale components, biochar-based nanocomposites (BCNs) exhibit markedly improved electrochemical behaviour arising from synergistic interactions between the porous carbon framework and functional nanomaterials. This review critically examines recent progress in BCNs for energy storage and conversion applications, including supercapacitors, lithium- and sodium-ion batteries, electrocatalysis and photocatalysis. Emphasis is placed on correlating biomass feedstock selection, pyrolysis and activation conditions. The nanocomposite architecture, with dominant charge-storage mechanisms such as electric double-layer capacitance, pseudocapacitive redox processes and ion intercalation, has been discussed. Reported BCNs demonstrate specific capacitances exceeding 400–700 F g^−1^, energy densities approaching 90 Wh kg^−1^, and long-term cycling stability beyond 10^4^ cycles, depending on composition and electrolyte environment. Beyond performance metrics, this review highlights theoretical study, key limitations related to feedstock variability, conductivity, and scalability. Emerging strategies, including heteroatom doping, hybrid architectures, and AI based data-assisted material optimization have been investigated. By integrating energy storage and conversion within a unified framework, this work provides a structured perspective on the rational design of scalable and sustainable biochar-based nanocomposites for next-generation energy technologies.

## Abstract 2

Advanced biochar nanocomposites is the future of low-cost and efficient energy storage materials.

## Graphical abstract

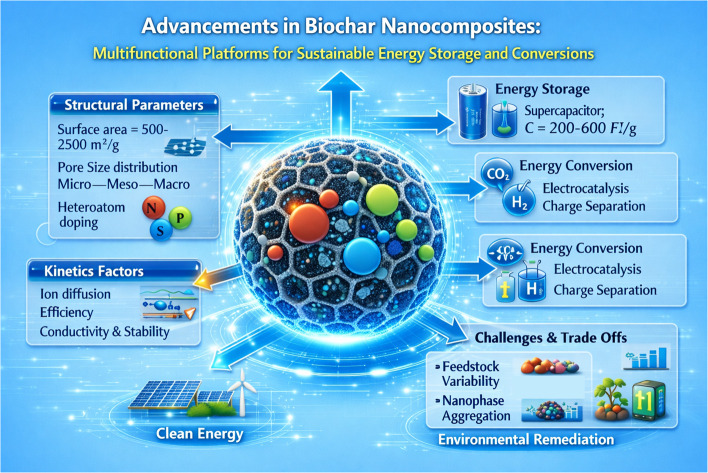

## 1. Introduction

The global energy paradigm is undergoing a profound transformation, driven by the urgent need to mitigate climate change and transition from fossil fuels to renewable sources. While solar, wind, and hydroelectric power offer a path toward sustainability, their inherent intermittency creates a critical disconnect between energy supply and demand.^1^ This disconnect necessitates the development of advanced, reliable, and efficient energy storage and conversion systems to stabilize the grid and power the next generation of portable electronics and electric vehicles.^2,3^ The performance of systems such as lithium-ion batteries (LIBs), supercapacitors, or fuel cells is intrinsically linked to the properties of their constituent electrode materials. Ideal materials must exhibit high electrical conductivity, large specific surface area, tailored porosity, and electrochemical stability, and be derived from sustainable, low-cost sources.

Supercapacitors are high-performance electrochemical energy storage devices that bridge the gap between conventional capacitors and batteries, offering exceptionally high power density, rapid charge–discharge rates (within seconds), and outstanding cycling stability (often exceeding 10 000–100 000 cycles). Unlike batteries, they store charge primarily through electrostatic ion adsorption at the electrode–electrolyte interface (electric double-layer capacitance) or fast surface redox reactions (pseudocapacitance), enabling superior power delivery while maintaining reasonable energy density.^4^ Flexible supercapacitors represent a transformative advancement by incorporating bendable substrates, gel or solid-state electrolytes, and mechanically robust electrode materials, allowing the devices to endure repeated deformation such as bending, folding, twisting, or stretching without significant loss in electrochemical performance.^5^ These attributes make them ideal for powering wearable electronics, smart textiles, and portable devices. Complementing them, micro-supercapacitors (microcapacitors) with miniaturized, on-chip planar architectures and interdigital electrodes enable seamless integration into microelectronic circuits and Internet-of-Things (IoT) sensors.^6^ Together, flexible supercapacitors and micro-supercapacitors are poised to become key technologies of the future, particularly when fabricated using sustainable, low-cost materials like biochar nanocomposites, offering environmentally friendly, high-power solutions for next-generation flexible and wearable energy storage systems.

**Fig. 1:**
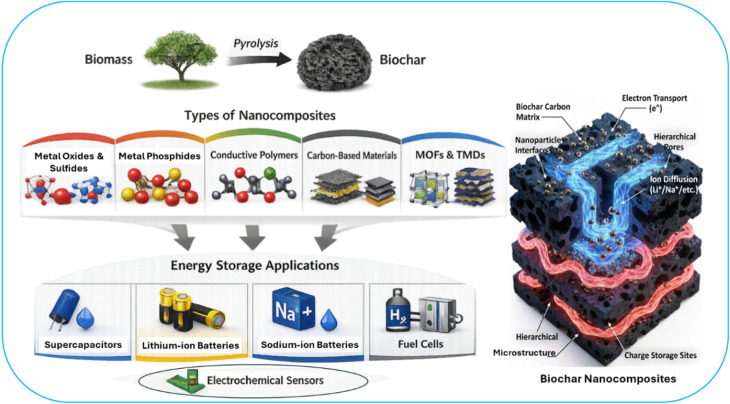
Different biochar nanocomposites for energy storage applications.

In this context, carbonaceous materials have become indispensable. They serve as conductive frameworks, structural supports that prevent electrode degradation, and hosts for ion intercalation or adsorption. Among the vast carbon family, biochar (a carbonaceous material produced from biomass pyrolysis) has emerged as a promising material. Biochar abundance, renewability and inherent porous structure provide a strong foundation for energy storage applications.^7^ However, despite these advantages, pristine biochar faces fundamental limitations that hinder its direct application in high-performance energy devices. Its carbon structure is often heterogeneous and poorly graphitized, resulting in modest electrical conductivity compared to engineered carbons like graphene or carbon nanotubes (CNTs).^8,9^ The integration of nanomaterials into biochar matrices has led to the development of multifunctional platforms that enhance electrochemical performance, conductivity and energy storage capacity.^10^ Furthermore, its physicochemical properties, such as porosity, surface functionality, and graphitic order, can be modulated by controlling the pyrolysis conditions (temperature, heating rate, residence time) and the choice of biomass feedstock. Researchers have developed materials with enhanced conductivity, higher surface areas and superior energy storage capabilities by exploring the synergistic effects between biochar and nanoscale additives.^11^ These advancements position biochar nanocomposites (BCNs) as a versatile, cost-effective and sustainable solution for next-generation energy storage devices.^12^

dos Reis *et al.* investigated different biomass physical characteristics (charging–discharging, electrode–electrolyte interfacing and electrochemical metrics) compatible with nanomaterials composition for high energy storage.^13^ New rational approach synergistically integrates nanoscale functional materials, such as metal oxides (*e.g.*, ZnO, TiO_2_, Fe_3_O_4_, MnO_2_), conductive polymers (*e.g.*, polyaniline, polypyrrole), carbon allotropes (graphene, CNTs), transition metal dichalcogenides (*e.g.*, MoS_2_), or MXenes into the porous biochar matrix for energy storage, batteries and supercapacitors.^11–17^ MnO_2_/BCNs are effective in pseudocapacitors due to their high specific capacitance.^18^ Fe_3_O_4_/biochar has been applied in lithium-ion batteries (LIBs) and magnetic energy storage.^19^ Metal-sulfide-BCNs, such as MoS_2_/biochar, have been widely used in supercapacitors for high energy density, and CoS_2_/biochar enhances electrochemical performance in batteries.^20^ Biochar/graphene can enhance conductivity and surface area in supercapacitors, while biochar/carbon nanotubes (CNTs) improve charge transfer and ion transport in energy storage systems.^10,21^ Biochar/activated carbon has been used in hybrid supercapacitors. Similarly, polymer-BCNs such as polyaniline (PANI)/biochar have been known to exhibit pseudocapacitance in supercapacitors. Polypyrrole (PPy)/biochar enhance energy storage performance due to improved conductivity.^18^ Metal–organic framework (MOF)-derived biochar composites offer high porosity and excellent electrochemical performance in energy applications.^22^ Silicon embedded in biochar improves energy storage capacities, particularly in LIBs. Pristine MXene (*e.g.* Ti_3_C_2_) suffers from interlayer restacking driven by van der Waals interactions and surface termination-induced oxidation, leading to reduced ion-accessible surface area and sluggish charge transfer. Biochar integration into MXene functions as a structural spacer that expands interlayer distance, improves electrolyte penetration, and enhances electronic conductivity.^23^ BCNs based sodium-ion batteries (SIBs) are an alternative to LIBs with cost-effectiveness. The conductive and porous properties of biochar are effective for fuel cell and electrochemical sensing.^24,25^Fig. 1 represents different energy storage applications and the complex electron/ion transport paths within BCNs.

Prior reviews have typically confined their scope to a single class of materials, a specific synthesis technique, or a singular application, such as biochar for supercapacitors. A critical gap remains in providing a holistic, integrated perspective that connects the entire materials chain—from the selection of biomass feedstock and optimization of pyrolysis conditions, through the rational design of the nanocomposite architecture, to the final multifunctional performance in both energy storage and conversion devices.^21^

This review aims to fill that gap by offering a comprehensive and critical examination of the field of biochar nanocomposites. Our approach is structured to provide a “synthesis-structure–properties–performance” roadmap. We have systematically explored how the choice of feedstock and pyrolysis parameters dictates the fundamental properties of the pristine biochar, and how these properties subsequently influence the design and function of the final nanocomposite. A central theme will be the correlation between BCN architecture and the dominant charge-storage mechanisms – EDLC, pseudocapacitance, and diffusion-controlled intercalation – providing a framework for understanding and predicting electrochemical behavior.^8,19,26^ We have critically evaluated the reported performance of BCNs in key application areas: supercapacitors, lithium-ion batteries, sodium-ion batteries, and electrocatalysis (specifically for the oxygen reduction reaction). Beyond a mere compilation of performance metrics (*e.g.*, specific capacitances exceeding 700 F g^−1^, energy densities approaching 90 Wh kg^−1^, and cycling stability beyond 10 000 cycles), we will contextualize these achievements within the fundamental electrochemical principles at play.

Furthermore, this review distinguishes itself by addressing the critical challenges that impede the practical translation of BCNs from the laboratory to commercial reality. We will delve into the issues of feedstock variability, which leads to inconsistent material properties; the trade-offs between enhancing performance and maintaining a simple, scalable synthesis; and the inherent conductivity limitations of biochar-derived carbons. In response to these challenges, we have highlighted cutting-edge, forward-looking strategies, including:

• Heteroatom doping: the intentional introduction of elements like nitrogen, sulfur, or phosphorus into the carbon lattice to modulate its electronic structure and create additional active sites for both charge storage and catalysis.

• Hybrid architectures: the design of nanocomposites that harness the complementary strengths of multiple nanoscale components.

• Data-assisted material optimization: the emerging role of machine learning and artificial intelligence in navigating the vast experimental parameter space to predict high-performance BCN formulations and accelerate the discovery process.

Finally, we have discussed the path forward, outlining key future directions for research and development focused on achieving scalability, ensuring environmental sustainability through life-cycle assessment, and ultimately enabling the commercialization of these promising multifunctional platforms. By integrating the domains of energy storage and conversion within a unified framework, this review provides a structured perspective essential for the rational design of next-generation, sustainable energy technologies.

## 2. C-polymorphs in biochar derived energy systems

Biochar-derived carbons consist of disordered sp^2^ and sp^3^ clusters embedded within amorphous matrices. The synthesis of biochar-derived carbon polymorphs is governed by input variables, including precursor chemistry, thermal evolution, activation strategy, catalytic effects, and heteroatom incorporation. Rational control of these parameters enables targeted engineering of hard, turbostratic or graphitic domains tailored to ion-specific storage mechanisms and charge-transfer requirements.^27^

**Fig. 2:**
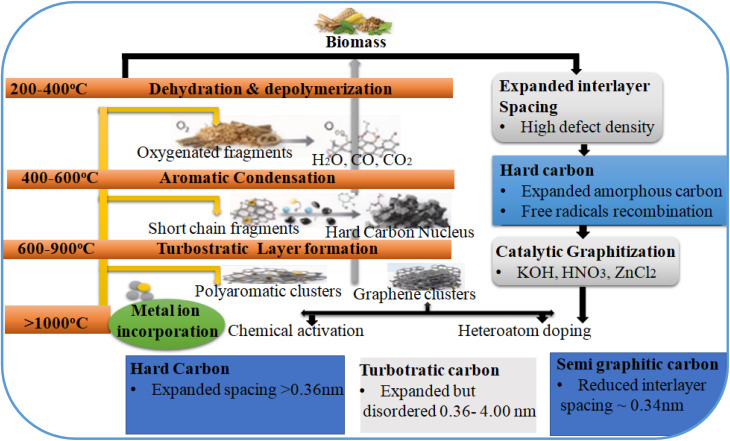
Schematic for synthesis of C-polymorphs in biochar derived energy systems and scheme of different biochar nanocomposites for energy storage applications.

The schematic illustration in Fig. 2 shows a multistage transformation from biomass precursors to distinct carbon polymorphs during thermochemical conversion and BCNs storage applications. The process begins with dehydration and depolymerization of biomass macromolecules (200–400 °C), which is followed by aromatic condensation and formation of polyaromatic clusters (400–600 °C). Progressive π–π stacking of graphene-like fragments produces turbostratic carbon with expanded interlayer spacing (600–900 °C). Higher temperatures (>1000 °C) can enhance atomic mobility, which promotes partial graphitization and reduction of interlayer spacing toward ∼0.34 nm.^28^ Catalytic graphitization *via* transition metals such as Fe, Ni, and Co can proceed *via* a dissolution precipitation mechanism, which accelerates ordered domain formation. Chemical activation, like KOH and ZnCl_2_, induces lattice expansion and micropore generation *via* intercalation and etching reactions. Heteroatom doping, such as nitrogen incorporation, can introduce lattice distortions and electronic modulation. The final carbon polymorph hard carbon, turbostratic carbon, or semi-graphitic domains, are the result of precursor chemistry, thermal conditions, catalytic effects and post-treatment strategies.^29^

Hard carbon is characterized by disordered turbostratic stacking, expanded interlayer spacing (>0.37 nm), a defect-rich framework, and closed nanopores. This structure is particularly advantageous for sodium-ion batteries (SIBs), where the larger Na^+^ ionic radius (1.02 Å) requires expanded diffusion pathways. Sodium storage often proceeds *via* adsorption pore filling mechanisms combined with limited intercalation. Turbostratic carbon consists of stacked graphene-like layers that are misaligned or may be rotationally disordered. The layers are parallel but lack the perfect AB stacking found in graphite.^30^ Turbostratic carbon contains an interlayer spacing that typically ranges from 0.36 to 0.40 nm, moderate conductivity, balanced defect density and short-range ordering. These characteristics are suitable for supercapacitors and hybrid battery systems. The expanded interlayer spacing improves accessibility, particularly for Na^+^ storage. Turbostratic domains provide an optimal compromise between structural disorder with active sites and electronic continuity as charge transport.^31^ Biochar can rearrange into semi-graphitic structures at elevated temperatures or under catalytic conditions, which can reduce interlayer spacing (∼0.34 nm). Semi-graphitic structures exhibit increased crystallite size, higher electronic conductivity, and lower defect density. Graphitic domains enhance electron percolation pathways and improve rate performance in high-power devices. However, excessive ordering may reduce the number of adsorption sites and limit Na^+^ intercalation due to a narrower interlayer spacing. Hard carbon is particularly effective for Na^+^ ion batteries due to its ability to accommodate Na^+^ through adsorption and pore filling mechanisms in addition to limited intercalation. Soft carbon (semi-graphitic structures) benefits Li^+^ ion storage and high-rate supercapacitor applications where rapid electron transport is essential.^32^

## 3. Tailoring approaches of BCNs

Biochar is derived from various biomass sources, resulting in distinct types with unique attributes and compositions. These differences arise from the type of feedstock and the pyrolysis conditions. For example, derived nanoporous biochar from chemically active (ZnCl_2_ and KOH) Norway spruce bark biomass.^33,34^ The prepared biochars have superior capability to store charge ions like Li^+^ and Na^+^. Li^+^ and Na^+^ have superior storage in nanoporous biochar because their small ionic radii (Li^+^ = 0.76 Å and Na^+^ = 1.02 Å), monovalent charge, low diffusion barriers, and compatibility with defect-rich disordered carbon enable fast, reversible adsorption/intercalation and stable solid electrolyte interphases formation, unlike larger or multivalent ions.^35^ BCN-based anodes feature hierarchical pore architecture, with micropores and mesopores integrated within a macroporous network, resulting in a large specific surface area. They offer favourable conditions for rapid charge ion transport. The presence of interconnected macropores shortens diffusion pathways and enhances electrolyte penetration, while mesopores provide efficient ion-transport channels to electrochemically active regions. Simultaneously, a high surface area, associated with abundant mesoporosity, increases the number of accessible active sites that can enable both reversible ion adsorption and effective intercalation.^36,37^ This synergistic pore structure supports substantial pseudocapacitive contributions, maintains high capacity under fast charge–discharge conditions and alleviates mechanical stress arising from volume changes during repeated cycling. Consequently, fundamental studies focused on tailoring surface area, pore volume and pore size distribution. Data matched to the dimensions of solvated Li^+^ and Na^+^ ions, which are essential for developing BCN materials with enhanced electronic conductivity, rapid electrochemical kinetics and long-term structural stability.^38^

**Fig. 3:**
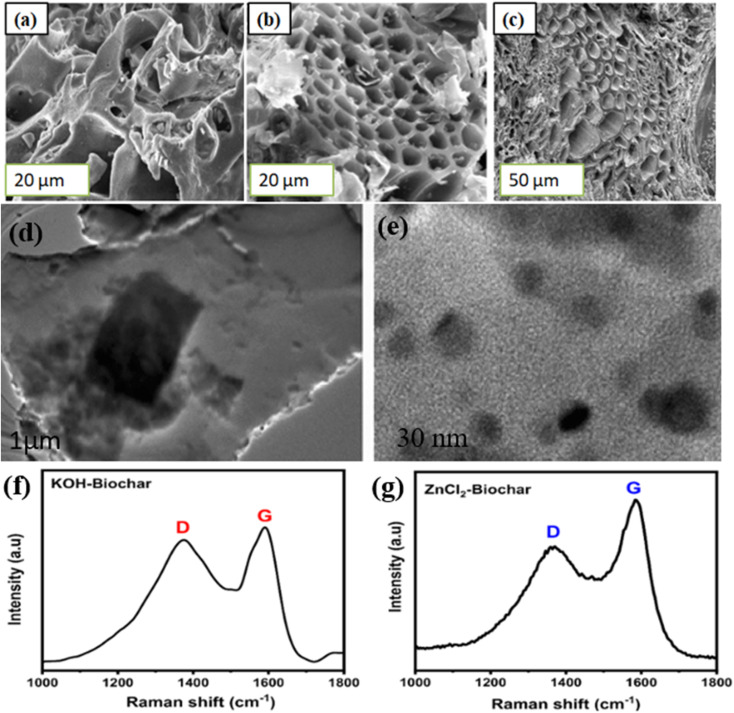
(a) SEM image of pine sawdust biochar^42^ adapted from ref. 42*via* Creative Commons CC BY 4.0 licence, [Springer Nature], copyright 2023 (b) SEM image of coconut biochar^43^ adapted from ref. 43*via* Creative Commons CC BY 4.0 licence, [MDPI], copyright 2021 (c) SEM image of barley straw biochar^44^ adapted from ref. 44*via* Creative Commons CC BY 4.0 licence, [Springer Nature], copyright 2025 (d and e) TEM image of CuO/Sea algae extracted biochar composite^45^ adapted from ref. 45*via* Creative Commons CC BY 4.0 licence, [Springer Nature], copyright 2023 (f and g) Raman spectroscopy of biochar samples showing different *I*_D_/*I*_G_ intensity ratio^46^ adapted from ref. 46*via* Creative Commons CC BY 4.0 licence, [MDPI], copyright 2025.

Pine sawdust biochar exhibits a highly porous and sponge-like structure, as seen in the SEM image Fig. 3(a).^39^ Generally, this biochar has a moderate surface area with a significant proportion of oxygen-containing functional groups, interconnected micro and mesopores. Coconut biochar has a denser, more rigid carbon structure than softwood biochar. SEM image Fig. 3(b) shows irregular, fractured carbon layers with micropores and some mesopores. Generally, coconut biochar has a high fixed carbon content and a relatively higher surface area than sawdust biochar and hard biomass, which results in a more stable and robust carbon matrix. FESEM image of Acacia leucophloea wood sawdust based biochar (Fig. 3(c)) shows uniformly distributed pores with smooth pore walls. Microstructure indicates well-developed microchannels formed during biomass decomposition, containing carbon-rich, aromatic structures typical of hardwood-derived biochars and good thermal stability due to the hardwood origin. The fabrication of BCNs involves several carefully designed techniques aimed at enhancing the functionality of energy storage systems. These methods ensure the optimization of the suitable structural, electrical and chemical properties of the materials for advanced applications.^40^ The initial step in biochar production (pyrolysis) involves the thermal decomposition of biomass in an oxygen-limited environment.^41^ The pyrolysis temperature, heating rate and type of biomass determine the material's properties. Table 1 shows biomass type, pyrolysis temperature, activation strategy, dopant/nanophase, surface area, electrochemical performance, limiting factors. Activation processes (chemical or physical) are often employed post-pyrolysis to introduce or enhance porosity.

**Table 1:** Comparison of biochar nanocomposite synthesis protocols and electrochemical performance

Biomass type	Pyrolysis temperature (°C)	Activation strategy	Dopant/Nanophase	Specific surface area (m^2^ g^−1^)	Electrochemical performance	Limiting factors	Ref.
Agricultural waste (*e.g.*, rice husk)	700–900	KOH chemical activation	N-doped carbon/MnO_2_	1200–1800	250–400 F g^−1^ (supercapacitor)	Possible pore collapse at high activation levels	47
Lignin-derived biomass	600–800	CO_2_ or steam activation	N,S co-doped carbon	800–1500	200–350 F g^−1^	Lower conductivity if graphitization is insufficient	48
Wood biomass	700–900	KOH or ZnCl_2_ activation	Metal oxides (MnO_2_, Co_3_O_4_)	900–1600	300–500 F g^−1^	Nanoparticle agglomeration at high loading	49
Coconut shell	800–1000	KOH activation + thermal treatment	N,P-doped BCN	1000–2000	Energy density 20–40 Wh kg^−1^	Higher processing temperature increases cost	40
Sugarcane bagasse	600–850	H_3_PO_4_ activation	Metal sulfides (NiS, CoS)	700–1400	250–450 F g^−1^	Structural degradation during long cycling	50
Plant leaf biomass	650–850	KOH + N-doping	MnO_2_ or Fe_3_O_4_	900–1700	150–300 mAh g^−1^ (hybrid battery)	SEI instability in battery configurations	47

Chemicals like KOH or H_3_PO_4_ are generally employed to enhance the specific surface area.^51^ Through *in situ* synthesis, nanoparticles, such as transition metal oxides or sulfides can be directly incorporated into biochar during biochar production. Mixing precursors with biomass before pyrolysis allows the particles to form uniformly within the biochar matrix.^9^ This method also provides a vigorous interaction between components and increases the stability and electrical traits of synthesized hybrid materials. Additional modifications can be applied *via* post-synthesis functionalization procedure to improve the performance of BCNs. Techniques such as doping with elements (nitrogen or sulfur) can increase conductivity, or coating the surface with polymers, such as polyaniline (PANI) can improve energy storage capacity.

These modifications introduce functional groups and active sites essential for electrochemical reactions. In template-guided fabrication, a sacrificial template during synthesis has been used to provide control over the pore structure of BCNs.^52^ Templates such as silica or polymer beads create specific patterns or hierarchies that can be removed post-synthesis, leaving highly ordered porosity that enhances ion transport and accessibility.

**Fig. 4:**
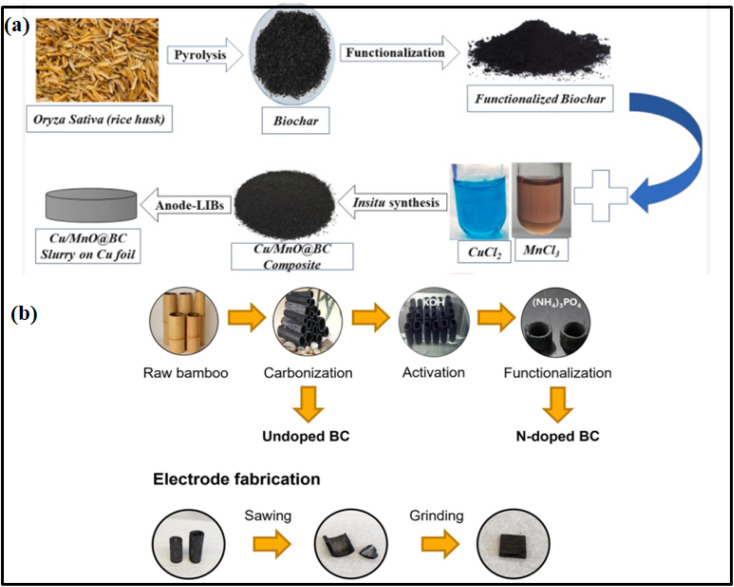
Schematic of synthesis of (a) rice husk biochar Cu/MnO based nanocomposite^58^ adapted from ref. 58*via* Creative Commons CC BY-NC-ND 4.0 licence, [Elsevier], copyright 2024 and (b) raw bamboo N-doped biochar for electrode preparation^59^ adapted from ref. 58*via* Creative Commons CC BY 3.0 licence, [Royal Society of Chemistry], copyright 2024.

The synthesis of BCNs involves integrating biochar with nanomaterials *via* various methods, including *in situ* growth, physical mixing, chemical functionalization, and hydrothermal and co-precipitation approaches. The choice of biomass feedstock, pyrolysis conditions, and nanomaterial type significantly influences the properties of the resulting nanocomposites. The developed corn straw biochar has a high surface area (2790.4 m^2^ g^−1^) and pore volume (2.04 cm^3^ g^−1^) for supercapacitor applications.^9^ Although porosity enhances ion transport, the biochar-derived carbon may still require additional treatments (*e.g.*, graphitization, doping, or conductive additives) to achieve higher electronic conductivity. Nanocomposites of metal/metal oxides (*e.g.*, Co, Ni, Fe, and Mn oxides) with biochar significantly increase specific capacitance and energy density due to synergistic effects between the pseudocapacitive metal oxides and the high surface area and porous structure of biochar. The symmetric devices assembled from RAB (Raw algal biochar), 3DFAB (3D interconnected mesopores network), and CoTLM (Tile-like microstructure containing cobalt oxides) deliver high specific capacitances, with CoTLM reaching 445 F g^−1^. The asymmetric configuration (3DFAB/CoTLM) exhibits an even higher capacitance of 411 F g^−1^, demonstrating effective utilization of both electrode materials.^40^ Although unbuffered aqueous electrolytes improve safety, they can impose restrictions on the voltage window and ionic conductivity compared to organic or hybrid electrolytes, potentially limiting the ultimate energy density. Biochar's inherent porous structure and high surface area provide ample active sites for electrochemical reactions and ion storage. Some metal oxides (such as MnO_2_, Fe_2_O_3_) have poor electrical conductivity, which can limit rate performance unless compensated by conductive additives or hybridization. Repeated faradaic reactions can cause structural degradation, phase changes, or volume expansion in metal oxides, leading to reduced long-term stability.^53^ The incorporation of conductive nanomaterials enhances the overall conductivity of the composites. BCNs with conductive materials such as metals, metal oxides, graphene, CNTs, and carbon quantum dots (CQDs) have been investigated for applications in supercapacitors and other energy storage devices.^54^ However, current research does not support their use as superconductors, which require zero electrical resistance at low temperatures. Nanocomposites of biochar exhibit improved mechanical strength, making them suitable for long-term applications. Advanced synthesis methods, such as template-assisted carbonization and chemical activation, yield hierarchical porous structures and multilayered nanocages. For example, mechanical durability and electrochemical performance can be improved by incorporating nanofibers into biochar.^55^ BCNs demonstrate high specific capacitance (up to 675 F g^−1^), such as Ag-biochar 517 F g^−1^ for exfoliated biochar, and 407 F g^−1^ for F-treated wood biochar and energy densities suitable for practical supercapacitor use.^56^ The synergistic effects of biochar's porous structure and the incorporation of nanomaterials, such as MnO_2_, Ag, FeS_2_, and WO_3_, can enhance ion transport, conductivity, and charge storage, supporting both high power and energy densities. These materials consistently show excellent cycling stability, with capacitance retention rates of 85–99.5% after 10 000–60 000 cycles, indicating strong long-term operational reliability.^57^ Functional groups on biochar can be modified to enhance interactions with nanomaterials and improve performance *via* tunable surface chemistry. Fig. 4(a) and (b) show a schematic of the synthesis of rice husk biochar Cu/MnO based nanocomposite^58^ and raw bamboo N-doped biochar for electrode preparation,^59^ respectively. Functional groups on biochar (such as oxygen, nitrogen, sulfur, and fluorine-containing moieties) can be introduced *via* chemical activation (*e.g.*, KOH, HNO_3_, H_2_SO_4_), heteroatom doping, or surface treatments.^60^

These modifications increase the density of active sites, improve wettability, and facilitate stronger interactions with nanomaterials.^38^ Green synthesis routes, such as using plant extracts, algae or low energy mechanochemical methods, are gaining traction for their sustainability and cost-effectiveness. Eltaweil *et al.* used *Chenopodium* ambrosioides leaf extract to prepare Ag-loaded biochar. BCNs with advanced synthesis and functionalization provide enhanced properties, highly effective for sustainable energy storage and conversion.^2^ Structural and morphological characterization techniques are utilized to evaluate tailored BCNs performance. *In situ* X-ray diffraction (XRD) provides complementary insight into interlayer spacing and phase evolution.^61^ In biochar-rich systems, limited *d*-spacing variation suggests capacitive dominance, whereas measurable lattice expansion implies partial ion insertion into hard carbon domains. For composites containing Mn-based phases, XRD can also detect reversible phase transitions during redox cycling. XPS or quasi-*in situ* surface analysis helps identify oxidation state changes in metal species and monitor interfacial charge redistribution between carbon and the active phase. Shifts in binding energy reveal electronic coupling, while stable carbon signals confirm the integrity of the framework. *In situ* transmission electron microscopy (TEM) provides direct nanoscale visualization of the structural evolution of biochar nanocomposites (BCNs) under electrochemical bias. Unlike *ex situ* imaging (Fig. 3d and e), which only captures post-cycling morphology, *in situ* TEM allows observation of dynamic processes such as lattice expansion, nanoparticle restructuring, defect propagation, and interfacial transformations in real time. Raman spectroscopy is widely used to quantify structural disorder. The D-band (∼1350 cm^−1^) corresponds to defect-induced breathing modes, while the G-band (∼1580–1600 cm^−1^) reflects in-plane graphitic vibration (Fig. 3f–g). If the intensity ratio (*I*_D_/*I*_G_) is higher, indicating increased disorder and defect density, while lower values correspond to greater graphitic ordering. Elevated *I*_D_/*I*_G_ often correlates with enhanced pseudocapacitive behaviour and Na^+^ storage. Lower *I*_D_/*I*_G_ values generally improve electrical conductivity and rate capability. Thus, Raman analysis provides a rapid structural descriptor that can be correlated with diffusion kinetics and charge-transfer resistance.

## 4. Theoretical and multiscale simulation of BCNs

Density Functional Theory (DFT) calculations offer insight into the adsorption behaviour of alkali metal ions like Li^+^, Na^+^ and K^+^ on biochar-derived carbon surfaces and at carbon nanophase interfaces. The ion adsorption energy (*E*_ads_) is typically defined in eqn (1), where a more negative value indicates stronger binding.^62^

1 *E*_ads_ = *E*_BCN+ion_ − *E*_BCN_ − *E*_ion_

Theoretical analyses include defect sites (vacancies, edges), expanded interlayer spacing and heteroatom doping analysis. Defect sites provide stronger adsorption than pristine basal planes, expanded interlayer spacing reduces diffusion barriers for Na^+^ and K^+^, and heteroatom-doped carbons such as N, S, and P significantly modify adsorption energetics. Li^+^ generally exhibits lower diffusion barriers due to a smaller ionic radius. Na^+^ adsorption benefits from defect-rich and expanded hard carbon domains, and K^+^ requires even larger spacing and open frameworks due to its larger ionic size. Thus, DFT enables ion-specific optimization of BCN structures.^63^ Electronic density of states (DOS) near the Fermi level modulation *via* heteroatom doping can reveal electronic conductivity and charge-transfer kinetics. In DFT simulations, graphitic-N increases the electron density near the Fermi level, potentially improving conductivity. Sulfur and phosphorus doping can induce lattice distortion, altering the band structure.^64^ An increased DOS at the Fermi level facilitates faster electron transfer between the carbon matrix and the active nanophase, directly reducing charge-transfer resistance. Therefore, theoretical DOS analysis provides quantitative insight into how doping strategies influence electrochemical kinetics.

For BCN-based electrocatalysts, the catalytic activity of supported metal nanoparticles/composites is influenced by electronic interactions with the carbon matrix. The D-band center (*ε*_d_) relative to the Fermi level governs the adsorption strength of reaction intermediates. An upward shift in *ε*_d_ increases adsorption strength, excessively strong adsorption may hinder product desorption, and optimal catalytic activity occurs at intermediate binding energies.^64^ Heteroatom-doped carbon supports can modulate the D-band center of anchored metal oxides or sulfides through charge redistribution at the interface. Thus, theoretical modelling provides a mechanistic justification for enhanced oxygen reduction reaction (ORR), hydrogen evolution reaction (HER) or oxygen evolution reaction (OER) performance in BCN systems.^65^

While DFT captures atomistic interactions, molecular dynamics (MD) simulations of ion diffusion enable investigation of ion transport within realistic pore architectures.^66^ It can calculate diffusion coefficients (*D*), ion trajectory pathways, solvation shell evolution and ion confinement effects in micropores. In hierarchical BCNs, mesopores act as fast ion highways, micropores contribute to adsorption but may slow diffusion if overly narrow, and tortuosity significantly influences ion mobility. Simulated diffusion coefficients can be correlated with experimentally measured rate capability and impedance spectra. Thus, MD provides a dynamic perspective on electrolyte pore interactions.

At the device scale, continuum modelling tools such as COMSOL multiphysics enable the simulation of ion concentration gradients, electric potential distributions, current density variations, and reaction kinetics within porous electrodes.^67^ Electrode scale models can integrate effective ionic conductivity, electronic conductivity, porosity, tortuosity, and reaction rate constants. Such simulations reveal how local ion depletion, overpotential distribution, and diffusion limitations impact practical capacitance and energy density. This approach bridges nanoscale properties with macroscopic device behaviour. The ionic flux (*J*) through porous carbon can be approximated by eqn (2), where *D*_eff_ depends on pore size distribution, connectivity, tortuosity and electrolyte viscosity.

2 *J* = −*D*_eff_∇*C*

Hierarchical pore systems reduce effective diffusion resistance by combining macropores as reservoirs, mesopores as transport channels, and micropores as adsorption sites. Theoretical modelling demonstrates that excessive microporosity, lacking transport channels, can reduce overall ionic flux despite a high surface area. Thus, optimal performance arises from balanced pore geometry rather than maximum surface area.^68^

## 5. Electrochemical properties of BCNs for next-generation storage devices and conversions

BCNs exhibit exceptional electrochemical properties, making them suitable for advanced energy storage devices. The observed performance improvements result from the interplay between the native structural attributes of biochar and the functional enhancements introduced by integrated nanomaterials.^69–74^ Upon activation, biochar develops a hierarchical porous network that significantly increases the available surface area for electrolyte ion adsorption.^75–78^ This feature is especially advantageous for supercapacitors, as charge storage in these systems primarily depends on electric double-layer formation rather than faradaic reactions.^36,37^ The combination of biochar with conductive nanomaterials, such as graphene, carbon nanotubes, precious metals or metal oxides, improves electron transport within the composite. The enhanced conductivity of the material leads to lower internal resistance and better charge–discharge efficiency.^33–35^

**Fig. 5:**
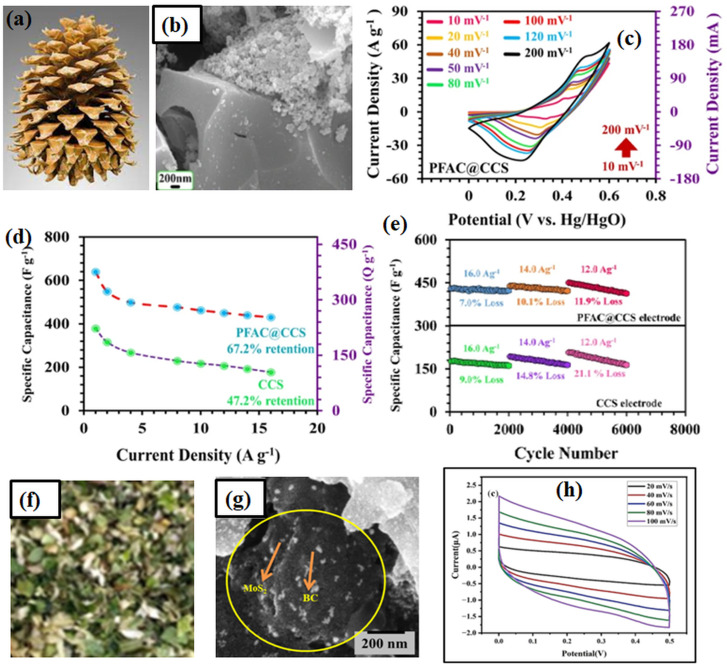
(a) Digital image of pine fruit (b) FESEM image of pine fruit char modified with CuCo_2_Se_4_ (c–e) high-performance battery-type supercapacitor (b–e)^39^ adapted from ref. 39*via* Creative Commons CC BY 4.0 licence, [Springer Nature], copyright 2025 (f) vegetable biomass (g) FESEM image of MoS_2_/biochar (h) MoS_2_/biochar performance for energy storage (g and h)^10^ adapted from ref. 10*via* Creative Commons CC BY 4.0 licence, [MDPI], copyright 2024.

BCNs with pseudocapacitive materials, such as MgO, MnO_2_ or Ni(OH)_2_, exhibit significant energy storage capabilities.^79^ These materials combine electrical double-layer capacitance with faradaic reactions to boost high energy density. The hierarchical pore structures in biochar facilitate efficient ion transport, diffusion and storage, minimizing diffusion resistance.^24,25^ This property enhances the rate capability and cycling performance of batteries and supercapacitors. Fig. 5(a) represents the pine fruit activated carbon modified with CuCo_2_Se_4_, which has higher performance for a battery-type supercapacitor than pure type biochar.^39^Fig. 5(b) represents the vegetable biomass for energy storage applications.^10^ BCNs demonstrate remarkable cycling stability on account of their structural robustness.^13,33–35^ Biochar nanomaterials maintain capacity over prolonged charge–discharge cycles in lithium-ion or sodium-ion batteries.^36–38^ The application of abundant biomass feedstocks for biochar production ensures economic feasibility and cost-effectiveness. BCNs offer a scalable solution for energy storage with the low cost of functionalization techniques. Table 2 explains the mechanism of various biochar based nanocomposites for energy storage. These electrochemical traits of BCNs define them as a promising material class for next-generation storage technologies. However, continued research into optimizing material properties and device integration will further improve their applicability in commercial energy storage systems.

**Table 2:** Mechanism of various biochar based nanocomposites for energy storage

S. N.	Mechanism	Biochar nanocomposites	Quantitative parameter (typical range)	Reactions	Characteristics/remarks	Ref.
1	Electric double-layer capacitive (EDLC)	Biochar/carbon-based composites such as biochar/graphene, biochar/CNTs	Specific surface area: ∼500–2500 m^2^ g^−1^	C^−^ + C^+^ ↔ (adsorbed ion pair on electrode surface)	Energy storage occurs through physical adsorption of electrolyte ions at the electrode surface	18
					Porous biochar provides a high surface area, enhancing charge storage capacity. No faradaic (redox) reaction occurs, making EDLC-based capacitors highly stable with fast charge/discharge cycles	
2	Pseudocapacitive	Biochar/metal oxides (MO) such as, biochar/MnO_2_, biochar/NiO, biochar/(Fe_3_O_4_)	Specific capacitance: ∼200–500 F per g (aqueous electrolytes)	MO + e^−^ + H^+^ ↔ MO(OH)	Energy storage occurs through reversible redox (faradaic) reactions involving metal oxides. Biochar improves electron transport and prevents nanoparticle agglomeration, leading to stable performance	80
3	Battery-type	Biochar/metal sulfides (MS) & phosphides such as biochar/MoS_2_, biochar/CoS_2_	Capacitance retention: ∼60–75% at ≥10 A g^−1^ reversible capacity (LIBs): ∼250–400 mAh per g (biochar); up to ∼600–900 mAh per g (BCNs)	MS + *x*Li^+^ + e^−^ + ↔ Li_*x*_MS	Such materials undergo conversion reactions where metal ions are intercalated and deintercalated within the structure. Biochar enhances conductivity and structural stability	81
4	Ion storage *via* intercalation	Biochar/Si or biochar/graphene oxide (GO) composites	Diffusion coefficient increase: ∼10–100× *vs.* dense carbons	C + Li^+^ + e^−^ ↔ Li_*x*_C	Energy storage occurs through ion intercalation into layered materials like GO. Biochar prevents volume expansion (such as Si anodes for lithium-ion batteries) enhancing cycle life	82
5	Hybrid energy storage	Biochar/polymers such as biochar/polyaniline (PANI), biochar/polypyrrole (PPy)	Interlayer spacing: ≥0.37 nm; capacity: ∼200–350 mAh g^−1^	Polymer + H^+^ + e^−^ ↔ polymer + (Protonated form)	Combines EDLC (biochar) and pseudocapacitive/battery-type (conducting polymers) mechanisms. Enhances capacitance and improves electrochemical stability	83
			Energy density ∼20–60 Wh kg^−1^ power density ≥5–10 kW kg^−1^ capacity/capacitance retention ≥90% after 10^4^ cycles			
6	Supercapattery	Biochar/MnO_2_/Mn_2_P_2_O_7_/Ni(OH)_2_/metal sulphides	Energy density ∼20–80 Wh kg^−1^ power density ≥1–10 kW kg^−1^ capacity/capacitance retention ≥90% after 5000 cycles	C + M^+^ + e^−^ ↔ C(M^+^)	Mixed kinetic control, higher energy than EDLC, reduced polarization, moderate internal resistance	84

To elucidate the charge storage kinetics of the BCN electrodes, cyclic voltammetry (CV) has been conducted over a broad range of scan rates (1–100 mV s^−1^, typically 5–10 incremental rates) for electrolyte and potential window employed for device evaluation. At any fixed potential, the measured current response arises from the combined contribution of surface-controlled processes (EDLC and fast surface redox reactions) and diffusion governed ion insertion into the bulk structure. To qualitatively distinguish these mechanisms, the relationship between current (*i*) and scan rate (*v*) has been analyzed using the power law expression as *i* = *av*^*b*^. After logarithmic transformation, log *i* = log *a* + *b* log *v*, the slope (*b*-value) provides insight into the dominant storage pathway: *b* ≈ 1 indicates a surface-controlled process, *b* ≈ 0.5 reflects diffusion-limited intercalation, and intermediate values signify mixed behavior. In the present system, pristine biochar exhibits *b* values close to unity, characteristic of ideal EDLC behavior, whereas incorporation of metal oxides or sulfides reduces *b* to ∼0.65–0.85, evidencing the emergence of pseudocapacitive and partial intercalation contributions.

For quantitative separation, the Dunn method was applied by expressing the current as *i*(*V*) = *k*_1_*v* + *k*_2_*v*^1/2^, where the term *k*_1_*v* corresponds to surface capacitive processes and *k*_2_*v*^1/2^ represents diffusion controlled charge storage. Rearranging to i/*v*^1/2^ = *k*_1_*v*^1/2^ + *k*_2_, linear fitting at selected potentials yields *k*_1_ (slope) and *k*_2_ (intercept). The fractional capacitive contribution at each scan rate was calculated from 
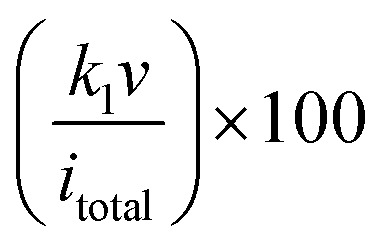
 and integrated across the entire potential range to generate capacitive and diffusion-controlled current distributions. Typically, at lower scan rates (≤5 mV s^−1^), diffusion related processes account for approximately 40–65% of the total response due to ion penetration into metal oxide lattices or sulfide layers. In contrast, at higher scan rates (≥50 mV s^−1^), the capacitive fraction exceeds ∼80%, dominated by the conductive biochar network and rapid surface redox reactions, thereby explaining the improved rate capability compared to pure biochar.

To further resolve surface and inner charge contributions within the capacitive domain, the Trasatti approach was employed by plotting stored charge against *v*^−1/2^, enabling estimation of outer (surface-accessible) and inner (diffusion-limited) charge components. This analysis confirms that, upon metal oxide/sulfide incorporation, the majority of the capacitive fraction originates from surface pseudocapacitance rather than purely EDLC effects. Overall, kinetic evaluation demonstrates that optimized BCNs achieve approximately 60–85% capacitive contribution at practical scan rates (10–50 mV s^−1^), indicating a favorable balance between energy density and power delivery. The combined *b*-value, Dunn, and Trasatti analyses provide a comprehensive mechanistic understanding of charge storage in these sustainable BCN-based hybrid systems and substantiate enhanced electrochemical performance.

## 6. Biochar derived carbon nanofiber for energy storage

Carbon nanofibers (CNFs) are one-dimensional carbon nanostructures with diameters typically ranging from 50–500 nm and lengths extending to several micrometers.^85^ Structurally, CNFs consist of stacked graphene layers arranged in different orientations such as platelet, ribbon, or herringbone structures. This arrangement provides CNFs with a combination of high electrical conductivity, mechanical strength, chemical stability, and tunable porosity. These features make CNFs particularly attractive for applications in energy storage devices, environmental remediation, catalysis and structural composites. Electrospinning is one of the most versatile and controllable techniques for producing CNFs.^86^ In this process, a polymer precursor solution is subjected to a high-voltage electric field, which generates a continuous jet of ultrafine fibers that are collected on a grounded substrate. Common polymer precursors include polyacrylonitrile (PAN), pitch, lignin and cellulose derivatives, which can be converted into carbon nanofibers through subsequent thermal treatments. The electrospinning process generally involves three major stages such as fiber formation, stabilization and carbonization. A polymer solution is electrospun under an electric field, producing nanoscale polymer fibers. Those fibers are heated in air at approximately 250–300 °C, where cyclization and crosslinking reactions occur, preventing fiber melting during carbonization. Stabilized fibers are heated in an inert atmosphere (typically nitrogen or argon) at 800–1000 °C, converting the polymer chains into graphitic carbon structures.^87^ Electrospinning offers precise control over fiber diameter, surface morphology, and pore distribution. By adjusting parameters such as solution viscosity, applied voltage, feed rate, and collector distance, researchers can tailor the structural properties of CNFs. Furthermore, functional materials such as metal oxides, metal nanoparticles, and heteroatoms (N, S, P) can be incorporated directly into the precursor solution, allowing the fabrication of multifunctional nanofiber composites. Another advantage of electrospinning is the formation of continuous nanofiber mats, which can serve as binder-free electrodes for electrochemical devices. The interconnected fibrous architecture facilitates rapid electron transport and efficient ion diffusion, both of which are critical for high-performance energy storage systems. Biochar-derived CNF hybrid materials are in demand due to increasing emphasis on sustainable and low-cost carbon sources. Biochar, produced through the pyrolysis of biomass such as agricultural residues, forestry waste, or plant materials, contains a porous carbon framework enriched with oxygen-containing functional groups. These features make biochar an excellent candidate for integration with carbon nanofibers. Biochar can contribute to CNF-based systems in several ways. First, it can act as a carbon precursor, where biochar particles are incorporated into polymer solutions during electrospinning. After thermal treatment, the resulting material forms a hybrid structure consisting of nanofiber networks embedded with porous biochar domains. This hierarchical structure significantly increases the available surface area and introduces additional adsorption or catalytic sites. Second, biochar can serve as a conductive support or reinforcement phase within CNF matrices. Such hybrid architectures have demonstrated promising performance in supercapacitors, electrocatalysis, and pollutant removal applications. Another important advantage of biochar-derived CNF hybrids is their environmental sustainability. Utilizing biomass waste as a carbon source reduces reliance on fossil-derived precursors and aligns with circular economy principles. Moreover, the presence of naturally occurring heteroatoms in biochar (such as nitrogen, sulfur, and oxygen) can enhance the electrochemical activity of the resulting nanocomposite. One of the key attributes of carbon nanofibers is their ability to significantly enhance the mechanical properties of composite materials.^88^ CNFs possess high tensile strength, elastic modulus, and flexibility, which enable them to act as effective reinforcement agents in polymeric, ceramic, and carbon-based matrices. When incorporated into composite systems, the fibrous structure of CNFs forms an interconnected network capable of efficient stress transfer. Under mechanical loading, stress is distributed along the nanofiber network, reducing localized deformation and preventing crack propagation. This mechanism improves both the strength and durability of the host material. In electrochemical devices, mechanical reinforcement is particularly important because repeated charge–discharge cycles can induce structural degradation.^89^ CNF frameworks help maintain electrode integrity, preventing particle aggregation or detachment from the current collector. This structural stability leads to improved cycling performance and long-term reliability. Furthermore, CNFs contribute to multifunctional reinforcement, meaning that they simultaneously enhance mechanical strength, electrical conductivity, and thermal stability. The conductive pathways formed by the nanofiber network facilitate rapid electron transport, which is beneficial for applications such as supercapacitors, batteries, and catalytic systems. The synergy between structural reinforcement and conductive functionality makes CNFs highly valuable for designing next-generation hybrid materials.^90^ When combined with porous carbons such as biochar or with active metal oxides, CNF-based composites can achieve a balance between mechanical robustness, high surface area, and efficient charge transfer, which are essential characteristics for advanced energy and environmental technologies (Table 3).

**Table 3:** Comparison of energy storage performance and properties of carbon nanofiber (CNF) hybrid structures

Material system	Structure type	Specific surface area (m^2^ g^−1^)	Specific capacitance/capacity	Energy density (Wh kg^−1^)	Power density (kW kg^−1^)	Cycling stability	Key advantages	Ref.
Pristine CNF	1D fibrous conductive network	100–400	100–250 F g^−1^	5–15	5–20	>90% after 5000 cycles	High conductivity, excellent rate capability	91
N-doped CNF	Defect-engineered CNF	200–500	200–350 F g^−1^	10–25	5–15	>92% after 5000 cycles	Enhanced pseudocapacitance, improved wettability	92
CNF/metal oxide (*e.g.*, MnO_2_)	Core–shell hybrid	150–450	300–600 F g^−1^	20–40	3–10	>90% after 5000 cycles	Redox-active sites, high capacitance	93
CNF/metal sulfide	Nanoparticle-decorated CNF	150–400	250–500 F g^−1^	25–45	3–8	>85% after 3000 cycles	High redox activity, improved energy density	94
CNF/biochar hybrid	Fiber–particle composite	400–1200	250–450 F g^−1^	15–35	5–15	>90% after 5000 cycles	Hierarchical porosity + conductive network	95
CNF/graphene hybrid	2D–1D conductive framework	500–1500	300–550 F g^−1^	20–40	5–15	>92% after 5000 cycles	High surface area and conductivity	96
CNF/conductive polymer	Polymer-coated CNF	200–500	350–700 F g^−1^	25–50	3–10	>85% after 3000 cycles	High pseudocapacitance and flexibility	97

## 7. Applications for energy storage devices and conversion

BCNs exhibit excellent specific capacitance and cycling stability on account of their hierarchical porous structures and hybrid compositions.^98,99^ The incorporation of biochar as an anode material in LIBs enhances capacity retention and charge–discharge rates. The structural tunability of biochar offers a cost-effective alternative to graphite in SIBs.

In lithium-based systems, Li^+^ ions (ionic radius ≈ 0.76 Å) readily insert into partially graphitized carbon domains when the interlayer distance meets or exceeds ∼3.35 Å. This process leads to the formation of staged intercalation compounds such as LiC_6_. Within BCN architectures, lithium diffusion coefficients fall within the 10^−9^–10^−10^ cm^2^ s^−1^ range, and structural expansion during cycling remains relatively modest (∼10%). Such limited volumetric strain supports long-term stability. To achieve this performance, a balanced carbon structure (Raman *I*_D_/*I*_G_ values typically between 0.9 and 1.1) and interconnected micro/mesoporous pathways are required to minimize transport resistance.^100^

Sodium storage needs a larger ionic radius (∼1.02 Å) since Na^+^ cannot reversibly form NaC_6_ within highly ordered graphite. Instead, charge storage in carbon anodes occurs through a combination of defect-site adsorption (often reflected as a sloping voltage profile), insertion into expanded graphene layers (requiring *d*_002_ values typically above 3.7 Å), and pore-filling contributions that generate low-voltage plateaus. Sodium diffusion in conventional carbons is significantly slower (approximately 10^−11^–10^−12^ cm^2^ s^−1^), and the associated lattice strain can reach 15–25%, increasing the risk of mechanical degradation.^101^ In BCNs, the interlayer spacing can be tuned between 3.45 and 3.95 Å, and the surface area (800–1800 m^2^ g^−1^). Integration with heteroatom-enriched defect sites reduces Na^+^ migration barriers. Density functional theory calculations indicate a decrease in the activation energy of roughly 0.3–0.6 eV, which improves rate performance, with capacity retention near 65% at 5 A g^−1^. N/P dual-doped biochar nanosheets and WS_2_ nanocrystal-embedded biochar further enhance sodium storage, offering high capacities up to 436 mAh g^−1^ and ultra-long lifespan up to 6000 cycles.^102^

Zinc-ion systems introduce additional complexity because Zn^2+^ carries a double positive charge and experiences stronger electrostatic interactions. Charge storage involves a combination of surface adsorption and redox conversion within supported metal oxides (*e.g.*, MnO_2_, which participates in reversible Zn^2+^-coupled reactions). The larger hydrated ion size and higher desolation energy necessitate mesoporous transport channels and mechanically resilient frameworks. Here, the disordered carbon matrix of BCNs, together with expanded interlayer spacing and strong C–O–M interfacial bonding, provides structural buffering that mitigates stress and prevents active material detachment. Post-cycling TEM analysis confirms preservation of morphology after extended Zn-ion cycling, and impedance evolution remains minimal. Electrochemical rate data further emphasize these ion-specific distinctions. At 5 A g^−1^, lithium systems retain approximately 78% of their capacity, sodium systems about 65% and zinc-ion configurations sustain stable output at moderate current densities due to their surface-redox-dominated kinetics. BCNs perform effectively in lithium systems with their intrinsically disordered structure and adjustable interlayer spacing. The heteroatom-rich chemistry also provides advantages for sodium and zinc storage.

Micro supercapacitors (µSCs) are planar, miniaturized energy storage devices designed for integrated electronics, wearable sensors, and Internet of Things (IOT) platforms.^6^ Unlike conventional stacked supercapacitors, µSCs utilize in-plane interdigitated electrodes that minimize ion diffusion distance and maximize power density within a limited footprint. BCNs have emerged as promising electrode materials due to their structural tunability, sustainability and compatibility with scalable fabrication methods. Biochar carbons exhibit hierarchical porosity, controllable defect density and adjustable graphitic ordering. These features are particularly advantageous for µSCs, where thin-film electrodes must simultaneously provide high areal capacitance and rapid ion transport. The electrochemical performance of µSCs is primarily evaluated using areal capacitance (mF cm^−2^) has shown in eqn (3). Where *I* is discharge current, Δ*t* is discharge time, *A* is active area, and Δ*V* is potential window. Therefore, maximizing accessible surface area within a confined electrode geometry is critical.3
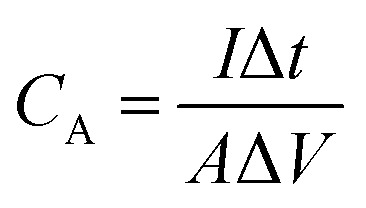


Laser-induced carbonization has become a powerful approach for producing conductive biochar electrodes directly on flexible substrates. Localized photothermal conversion promotes rapid aromatization, formation of semi-graphitic domains and development of interconnected porous networks.^103^ This mask-free, binder-free technique enables precise patterning of an interdigitated architecture while maintaining mechanical flexibility. Inkjet and screen printing are scalable alternatives for depositing BCN-based inks. Proper control of dispersion stability, viscosity, and particle size is essential to ensure uniform electrode formation. Post-print annealing improves interparticle contact and enhances conductivity without compromising porosity. µSCs typically employ planar interdigitated designs consisting of alternating electrode fingers. This configuration shortens ion diffusion pathways, reduces internal resistance, enhances power density and improves rate capability. Optimization of finger width, inter-finger spacing, and electrode thickness significantly influences ionic flux and electrochemical response. Excessive electrode thickness, although increasing active mass, may hinder diffusion and reduce performance rates. The carbon polymorph plays a decisive role in µSCs' behaviour. Amorphous carbon contributes surface functional groups and pseudocapacitance but suffers from limited conductivity. Highly graphitized domains enhance electron transport but may reduce active defect sites. Turbostratic carbon, characterized by moderate ordering and expanded interlayer spacing, often provides the optimal balance between conductivity and ion accessibility in µSC configurations.^104^ To further enhance performance, biochar is combined with redox-active nanophases such as MnO_2_, Ni(OH)_2_, Co_3_O_4_, or conductive polymers. In these hybrids, biochar provides a conductive scaffold, and electric double layer capacitance (EDLC) and metal oxides contribute surface redox reactions. Hierarchical porosity facilitates rapid ion diffusion.

However, excessive nanophase loading can block micropores and increase diffusion resistance. Controlled loading is therefore essential to preserve accessible surface area and maintain rate capability. BCN based µSCs can be integrated onto polyimide films, PET substrates, textiles and paper. Mechanical flexibility and structural resilience are essential for wearable electronics. Hierarchical porous networks help maintain conductive pathways under bending or stretching, enabling stable capacitance retention during repeated deformation cycles.^105^ Biochar-based nanocomposites provide a sustainable and structurally versatile platform for µSCs technology. By integrating optimized carbon polymorphism, hierarchical porosity and redox-active nanophases within interdigitated planar architectures. BCNs can deliver high areal capacitance, mechanical flexibility, and scalable fabrication potential, making them suitable for next-generation microscale energy systems.

BCNs are categorized based on their composition and the type of nanomaterials integrated.^106,107^ BCNs have also gained attention for their role in energy conversion applications, such as fuel cells, electrocatalysis and photocatalysis.^108^ BCNs are being investigated as catalysts and catalyst support in fuel cells. The synergistic combination of biochar with metal nanoparticles and catalytic materials has led to advancements in ORR, HER and photocatalytic conversions. Their high surface area and chemical stability make them ideal for ORR and HER. Functionalized biochar composites have shown performance comparable to that of traditional platinum-based catalysts at a fraction of the cost. Metal-doped (Fe, Co, Mn) and heteroatom-doped (N, S, P) BCNs significantly enhance the ORR, a key step in microbial fuel cells (µFCs), leading to higher power outputs.^109^ BCNs are also employed in electrocatalytic applications, such as water splitting and CO_2_ reduction. The synergistic effects between biochar and nanomaterials enhance catalytic activity and selectivity, enabling efficient conversion of renewable energy into chemical fuels. Heteroatoms doped biochar composites exhibit high catalytic activity. The high conductivity and stability of biochar enhance its use in hydrogen production for clean energy solutions. Functionalized biochar with metal oxides such as ZnO and TiO_2_ enables efficient solar-driven water splitting and pollutant degradation.^110,111^ Multicomponent metal oxides integrated within BCNs exploit synergistic redox interactions and electronic coupling to enhance charge storage. The disordered carbon framework, hierarchical porosity and tunable interlayer spacing maintain the electrochemical behaviour of biochar. BCNs contain short-range ordered graphene domains embedded in an amorphous matrix, creating multiple active environments for ion storage. In purely capacitive systems, charge is stored through electrostatic adsorption of electrolyte ions within accessible micropores and mesopores. The high internal surface area of biochar, combined with interconnected pore channels, allows rapid ion migration and short diffusion paths. This mechanism supports high power output and long cycling life as no structural phase change occurs during operation. Beyond surface adsorption, intrinsic defects and oxygen-containing functional groups introduce additional redox-active sites. These surface reactions contribute pseudocapacitance, especially when biochar is combined with transition metal oxides. The carbon matrix enhances electrical conductivity and stabilizes the redox-active phase, which allows fast and reversible charge transfer. In hard-carbon regions of biochar, expanded interlayer spacing enables partial ion insertion. Here, storage involves a combination of surface adsorption, interlayer insertion, and pore filling within nanovoids. Because these mechanisms coexist, biochar-based devices rarely behave as purely capacitive or purely battery-type systems. Instead, their position on a Ragone plot can be adjusted through structural tuning. Highly porous, defect-rich biochar favors high power density, whereas increased graphitic ordering or incorporation of redox-active phases shifts performance toward higher energy density. Thus, the multifunctional carbon architecture of biochar enables controlled movement across the energy–power spectrum, bridging conventional supercapacitors and battery-type storage systems.^112–114^ The utilization of biomass waste reduces carbon footprints and promotes circular economy targets. To scale up production for commercial energy demands, research must be focused on improving fabrication techniques and optimizing nanomaterial traits.^115,116^ Future advancements, such as artificial intelligence (AI) and generative AI driven materials design and optimization will further integrate biochar materials into next-generation energy storage and conversion systems. It will be a good progress towards a cleaner and more sustainable energy landscape. BCNs, incorporated in synergy with metal oxides such as MnO_2_, FeS_2_, WO_3_, NiO or doped with heteroatoms like N, S, P, serve as advanced electrodes. Such BCNs have achieved high specific capacitance (up to 699 F g^−1^) and energy densities (up to 94.5 Wh kg^−1^).^117^ These materials leverage biochar's porous structure for efficient ion transport and large surface area, while nanomaterial incorporation boosts conductivity and charge storage. BCNs are used in both symmetric and asymmetric supercapacitor configurations, enabling devices with high power and energy densities, rapid charge/discharge rates and long cycle life (often >90% retention after thousands of cycles). Asymmetric devices, pairing biochar-based electrodes with metal oxide or quantum dot composites, further enhance performance and operational voltage windows.^118^ Biochar composites are integrated into flexible, self-healing supercapacitors suitable for wearable electronics and low-temperature environments, maintaining high energy density and stability even at subzero temperatures. The use of agricultural and food waste as biochar sources supports eco-friendly, low-cost, and scalable supercapacitor production.^119^

The porous structure of biochar accommodates volume changes during ion insertion/extraction, reducing electrode degradation. Additionally, the incorporation of nanomaterials like silicon or tin oxides improves ion diffusion and conductivity. Chemically activated biochar, such as ZnCl_2_-activated with high mesoporosity and pyridinic-nitrogen groups, delivered excellent rate capability and long-term cycling stability (with capacities up to 370 mAh g^−1^ after 100 cycles and retention over 5000 cycles at high current densities).^13^ Biochar-CNT-NiO composites further boost reversible capacity up to 674.6 mAh g^−1^ after 100 cycles, outperforming biochar or NiO alone due to improved electron transfer and volume change mitigation.^120^ Chemical activation (ZnCl_2_, KOH) and heteroatom doping (N, P) increase porosity, surface area, and interlayer spacing, improving ion transport and storage. Incorporating metal oxides (NiO, Mn_3_O_4_, WS_2_) or CNTs enhances conductivity and mechanical stability while mitigating volume changes during cycling. Biochar is derived from renewable biomass, offering a green, cost-effective alternative to fossil-based carbons. Its use can reduce reliance on critical raw materials and lower the environmental footprint of battery production.^121^ Biochar's conductive, aromatic structure facilitates electron shuttling and storage, improving charge separation and reducing recombination in electrocatalytic reactions.^122^ Biochar supports uniform dispersion of metal or metal oxide nanoparticles such as CuWO_4_, Pd–Au, Fe_3_O_4_, Ni, and Ag, increasing the number of accessible active sites and boosting catalytic efficiency.^13,36–38,60^ BCNs offer a sustainable, high-performance platform for electrocatalysis, excelling in pollutant degradation, energy conversion, and sensing. Their tunable properties and synergistic effects with nanomaterials position them as promising alternatives to conventional catalysts.

## 8. Challenges and future prospects

Despite high potential and laboratory-scale performance, the translation of biochar-based nanocomposites into practical energy storage and conversion systems remains constrained.^123–125^ One of the most fundamental issues arises from biomass feedstock variability, which introduces significant dispersion in physicochemical properties. Experimental reports indicate that variations in cellulose, hemicellulose, and lignin content can alter biochar carbon yield by more than 20–30% and lead to surface area fluctuations exceeding ±40% under identical pyrolysis conditions.^57,98,99,106–108^ Such variability directly impacts electrochemical reproducibility, with reported deviations of up to 15–25% in specific capacitance or reversible capacity across batches, underscoring the need for feedstock pre-standardization or adaptive processing protocols.^126^

Porosity optimization presents another critical challenge when evaluated quantitatively. While activation can increase specific surface area beyond 1500–2000 m^2^ g^−1^, capacitance gains often saturate once accessible surface area exceeds ∼800–1000 m^2^ g^−1^.^110–114^ Beyond this threshold, excessive micropore formation (<0.7 nm) limits accessibility and increases internal resistance, particularly under high current densities. Electrochemical impedance studies consistently show that charge transfer resistance may increase by 30–50% in over-activated biochars, leading to reduced rate capability despite higher nominal surface area. Future efforts must therefore focus on pore size distribution control rather than surface area maximization.

The incorporation of redox-active nanophases introduces further quantitative trade-offs. While metal oxide or sulfide loading can enhance gravimetric capacity by 2–3 fold, loadings above ∼30–40 wt% frequently result in pronounced capacity fading, with retention dropping below 70% after 500 cycles.^57,60^ This degradation is primarily attributed to volume expansion, nanoparticle agglomeration and loss of electrical contact. Quantitative optimization of nanophase content, guided by percolation theory and interface resistance analysis, will be essential for achieving stable long-term performance. Kinetic limitations remain particularly significant in battery-type BCNs. Although hierarchical porosity can increase ion diffusion coefficients by one to two orders of magnitude compared to dense carbons, practical diffusion values remain in the range of 10^−12^–10^−10^ cm^2^ s^−1^ for Li^+^ and Na^+^ systems.^127,128^ These values are still lower than those required for high-power applications. In case of areal loadings should be relevant to commercial electrodes (>2 mg cm^−2^). Future research must therefore address ion transport at both particle and electrode scales, including electrode densification effects that are often neglected in laboratory studies.

Scalability and energy efficiency pose additional quantitative constraints.^129–131^ Many reported BCN synthesis routes involve activation temperatures above 800 °C and multiple chemical treatment steps.^129–135^ So, resulting in energy inputs that can exceed the embodied energy of conventional carbon materials. Preliminary life-cycle assessments suggest that unless activation severity and processing steps are reduced by at least 30–40%, the environmental advantage of biomass-derived carbons may be marginal. Developing low-temperature self-activation pathways and solvent-free nanocomposite fabrication strategies represents a critical future direction.

**Fig. 6:**
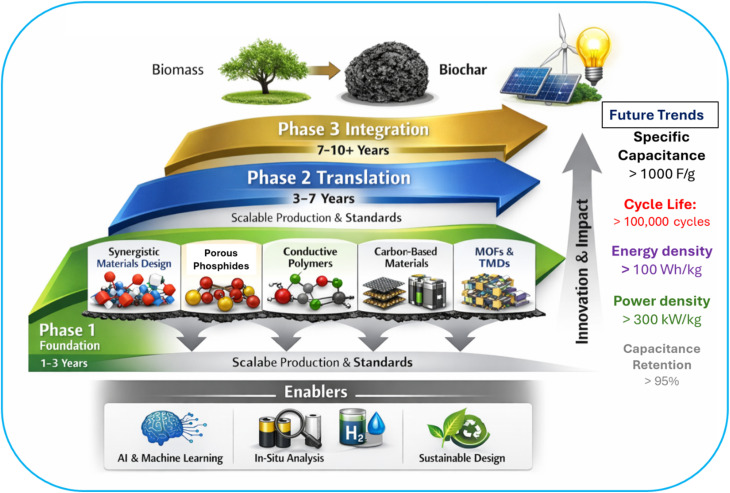
Roadmap for developing scalable, high-performance BCNs for energy storage.

From a performance benchmarking standpoint, the lack of standardized testing protocols complicates quantitative comparison across studies.^136–139^Fig. 6 presents a unified roadmap for developing scalable, high-performance, sustainable BCNs for energy storage. Differences in mass loading, electrolyte composition, voltage window and cycling protocols can lead to apparent performance variations exceeding 50%, even for structurally similar materials.^140–142^ Future work should prioritize reporting normalized metrics, including areal capacitance, volumetric energy density and energy efficiency under realistic operating conditions, to enable meaningful assessment of BCN performance. Looking forward, the future scope of biochar-based nanocomposites lies in data-driven and mechanism-guided material design. Integrating high-throughput experimentation with machine learning models trained on quantitative descriptors. Parameters such as pore size distribution, defect density and interfacial resistance could accelerate optimization while reducing experimental redundancy. Approaches such as coupling of mechanistic characterization techniques and techno-economic analysis can bridge the gap between laboratory performance and scalable, application-relevant energy technologies.

The optimization of BCNs through AI involves numerous correlated synthesis and structural parameters, such as pyrolysis temperature, activation chemistry, pore size distribution, heteroatom content, graphitization index, and nanophase loading input variables data sets.^4^ Generally, high-dimensional datasets have limited sample sizes, making them susceptible to overfitting when used directly in regression models. Principal Component Analysis (PCA) provides an effective preprocessing strategy by transforming correlated descriptors into orthogonal principal components that capture dominant variance. This dimensionality reduction mitigates redundancy and enhances model stability. PCA can supervise learning models such as Gradient Boosting Machines (GBM). Random Forest (RF) adoptive neuro (ANFIS) fuzzy logic can be employed to correlate structural descriptors with electrochemical outputs (capacitance, energy density, diffusion coefficient). Cross-validation ensures predictive reliability, while metrics such as RMSE and *R*^2^ evaluate regression accuracy. AI provides identification of dominant performance-governing descriptors, prediction of optimal synthesis windows and reduction of experimental redundancy. AI-driven BCN design thus transitions from empirical trial error to predictive, descriptor-guided optimization. AI enables rational optimization of hierarchical porosity, interfacial electronic coupling, and charge storage pathways, thereby accelerating the transition of BCNs from empirical materials to performance-engineered systems.

Developing standardized protocols for biochar synthesis and modification will improve reproducibility and facilitate industrial-scale production. Combining biochar with advanced nanomaterials such as MXenes, MoS_2_, and graphene can yield synergistic effects for next-generation energy storage. ML transforms BCN development from empirical optimization to data-driven inverse design by correlating synthesis parameters with electrochemical metrics. AI does not replace mechanistic understanding but complements it. When integrated with hierarchical structure engineering and interfacial electronic modulation, AI-driven optimization transforms BCN development from empirical material screening to predictive, descriptor-guided design. Although AI implementation in BCN research faces many challenges like limited and non-standardized datasets, inconsistent reporting of mass loading and volumetric metrics, variability in biomass feedstock composition and risk of over fitting in small datasets. Future research should focus on advancing low-cost, scalable synthesis methods, such as hydrothermal carbonization and eco-friendly activation techniques, to transform abundant biomass waste into high-performance electrodes for supercapacitors and batteries. Key priorities include enhancing electrical conductivity and electrochemical stability through innovative composites incorporating nanomaterials like graphene or carbon nanotubes, alongside surface functionalization with nitrogen-doping or oxygen-containing groups to boost pseudocapacitance and overall energy density. Addressing critical gaps such as long-term environmental impacts, carbon sequestration efficiency, and economic viability will require interdisciplinary research focusing on biomass-derived carbons, hierarchical porous structures, and multifunctional designs for flexible electronics, while aligning with circular economy principles to ensure widespread adoption in renewable energy systems by 2030 and beyond.

## 9. Conclusion

High-performance BCNs represent a promising class of materials for sustainable energy storage applications. Their tunable traits, derived from versatile synthesis strategies and hybridization with nanostructures, offer immense potential for enhancing energy density, stability and scalability. Despite their potential, BCNs face challenges such as feedstock variability, scalability and cycling stability. Biochar has inconsistent properties due to biomass origin and pyrolysis conditions. Hence, there is difficulty in large-scale production of uniform nanocomposites. Structural degradation of biochar over repeated cycles affects performance. Future research directions include optimization of synthesis techniques to develop scalable, eco-friendly processes, advanced characterization to utilize *in situ* techniques to understand charge storage mechanisms and integration in hybrid systems to combine BCNs with advanced energy storage technologies such as solid-state batteries, hybrid capacitors. Addressing current challenges will pave the way for their broader adoption in the energy storage industry, contributing to the realization of a sustainable energy future.

## Author contributions

Monika Dubey: conceptualization, investigation, methodology, writing – original draft, review and editing Piyush Kuchhal: review and editing, Ranjit Kumar: conceptualization, review writing and editing.

## Conflicts of interest

There are no conflicts to declare.

## Data Availability

No primary research results, software or code have been included, and no new data were generated or analysed as part of this review. All the data presented in this review paper have been duly cited in references.
